# Asthma in the elderly: the effect of choline supplementation

**DOI:** 10.1186/s13223-016-0121-5

**Published:** 2016-03-11

**Authors:** Michele Columbo, Albert S. Rohr

**Affiliations:** Division of Allergy and Immunology, Bryn Mawr Hospital, Bryn Mawr, PA USA

**Keywords:** Asthma, Choline, Elderly

## Abstract

**Background:**

Asthma in the elderly is poorly understood as very few studies have included these patients. DNA methylation can affect the expression of asthma susceptibility genes. Methyl groups can be produced through a choline dependent pathway. Asthmatics have decreased serum choline. We studied the effect of choline supplementation in elderly asthmatics and associations between different parameters at baseline.

**Methods:**

This is a double-blind, placebo-controlled, cross-over study. Thirty asthmatics 65 years old and older were evaluated at baseline and 3, 6, 9, and 12 weeks later. They randomly received choline bitartrate 310 mg and placebo capsules twice daily for 6 weeks.

**Results:**

Ninety percent of the study subjects were atopic and 97 % of them were using inhaled corticosteroids. Choline supplementation did not affect ACT (asthma control test), spirometric values, eosinophil counts or total serum IgE vs. placebo (p > 0.86 for all comparisons). In subjects with lower ACT (≤20), lower FEV1 % (<60 %), or higher eosinophil counts (≥0.6), there was similarly no difference between choline and placebo (p > 0.63). We found no significant association between eosinophil counts and IgE and the other parameters at baseline including in subjects with lower ACT or on higher inhaled steroid doses (p > 0.09). Asthmatic women had lower baseline ACT scores compared to men (p = 0.02).

**Conclusions:**

In this study of elderly asthmatics, choline supplementation for 6 weeks did not affect ACT scores, spirometric values, peripheral blood eosinophils, or total serum IgE. These results will require confirmation in larger and longer studies.

*Trial registration* ClinicalTrials.gov NCT02371993

## Findings

### Background

The U.S. population older than 65 years of age is growing rapidly and will likely increase to about 25 % of the total population by the year 2050 [1]. Among individuals 65 years old and older, about 7 % have asthma [1]. However, there is little information about asthma in these patients as most asthma studies have ignored this group. Older asthmatics have higher morbidity and mortality and are more likely to be underdiagnosed, undertreated, and hospitalized compared to their younger counterparts [1].

In elderly patients with asthma, we have previously studied the role of exhaled nitric oxide measurements and vitamin D [2, 3]. Indeed, there is growing interest in the possible role of dietary supplements (e.g., vitamins and methyl donors) in asthma [4, 5]. However, most of the studies of supplements in asthma have yielded conflicting or negative results [4, 5]. DNA methylation is a mechanism regulating gene-environment interactions in asthma, and its changes can affect asthma by increasing or decreasing the expression of asthma susceptibility genes [4, 5]. In humans, dietary methyl groups can be produced through folate and choline dependent pathways.

Choline is a water soluble essential nutrient important in neurotransmission, lipid signalling and membrane structure besides being a methyl donor [6]. It forms methionine through the methylation of homocysteine. Choline is contained in foods such as meat, liver, eggs, poultry, fish, shellfish and peanuts. Its deficiency has been associated with neurological and cardiovascular diseases [7]. Mostly without strong scientific evidence, choline supplementation is used for liver disease, neurological diseases including depression, bodybuilding, in pregnant women to prevent neural tube defects and as a supplement in infant formula.

In mice, choline has been shown to decrease airway allergic inflammation and reduce bronchoalveolar lavage eosinophils [8]. Individuals with asthma were found to have decreased serum choline [9]. An open label study of Indian asthmatic adults showed a decrease of bronchial hyperreactivity and use of asthma drugs after choline supplementation [10]. In the same study, there was a reduction of cytokine levels (IL-4 and TNFα) and cysteinyl leukotrienes in the supernatants of the patients’ blood mononuclear cells stimulated with phytohemoagglutinin [10]. In a recent, comprehensive review of vitamins and methyl donors in asthma, it was suggested that the current evidence strongly justifies clinical trials of choline supplementation as an adjuvant treatment in asthma [4].

The main purpose of this study was to investigate the effect of choline supplementation on asthma symptoms, assessed by the asthma control test (ACT), and spirometric values in elderly asthmatics. This is a double-blind, placebo controlled, cross-over study. Secondary objectives of our study included studying whether choline supplementation in elderly asthmatics affects peripheral blood eosinophil counts and total serum IgE levels. We also looked for associations between different parameters at baseline.

## Methods

This study included five study visits (baseline, and 3, 6, 9, and 12 weeks later). Each study subject took one capsule of choline bitartrate (310 mg) (Vitamin Shoppe, North Bergen, NJ) twice daily (total daily dose = 620 mg daily) or one placebo capsule twice daily each for 6 weeks in a double-blind, cross-over design. Individuals with history of gastrointestinal cancers were excluded from this study. The bottles containing the unused capsules were collected at the end of the two study periods, the remaining capsules counted and their number recorded. Compliance with the study based on capsule count was very good (>90 %).

Thirty subjects 65 years old and older with asthma were included in the study. Twenty-nine were Caucasian and one was African-American. Current smokers or individuals with a 10 pack/year or longer history of smoking were excluded. Almost all of the study subjects were lifetime nonsmokers. The study subjects were recruited among interested and eligible patients with asthma followed in our practice. Allergic sensitization was verified by allergy skin tests for relevant perennial and seasonal allergens.

Spirometric values were obtained according to the ATS/ARS guidelines by a KoKo Spirometer (nSpire Health, Inc, Longmont, Colorado, USA). The ACT is a tool that allows patients to report asthmatic symptoms on a scale from 1 (severe) to 5 (no symptoms) by answering five questions about asthma control. Values lower than 20 are considered as indicative of suboptimal asthma control. Inhaled steroid doses are expressed as fluticasone equivalent. The inhaled steroids used by the study subjects were fluticasone (17), budesonide (7), mometasone (4), and beclomethasone (1). Long-acting bronchodilators were salmeterol (12) and formoterol (8). The only leukotriene antagonist used by the study subjects was montelukast. Drug treatment remained essentially unchanged throughout the study period. One subject was on prednisone 5 mg daily throughout the study. Peripheral blood eosinophils were enumerated and total serum IgE measured at the Main Line Health Laboratories. This study was approved by the Main Line Hospitals Institutional Review Board (F/N-R15-3427B).

The primary endpoint of this study was to evaluate the effect of choline supplementation on the ACT scores. The goal was set to see a 10 % improvement (effect size) of the ACT score following treatment with choline vs. placebo. In our recent study of asthma in the elderly (2), the mean ACT score was 22.2 and the standard deviation was 2.8 % (n = 30). The calculated standardized effect size is 10 % of 22.2 (2.2)/2.8 = 0.8. Therefore, β = 0.2 (1/0.8). For an α of 0.05 (two tailed t test) the number necessary to see a 10 % change is 25. Data were expressed as the mean ± standard deviation and analyzed by the two tailed t test and the correlation coefficient as indicated with significance accepted at <0.05.

### Results

Table 1 summarizes the baseline characteristics of the study subjects. Most subjects were atopic (90 %), on inhaled corticosteroids (97 %), and had well controlled asthma. As shown in Table 2, choline supplementation for 3 or 6 weeks did not affect ACT or spirometric values when compared to placebo.

**Table 1:** Subjects’ characteristics at baseline

Sex (F/M)	18/12
Age (years, range)	73.7 ± 5.9 (66–84)
BMI	25.6 ± 4.7
Atopy	27/30
Duration of asthma (years)	34.8 ± 21.2
Rhinitis	23/30
Gastroesophageal reflux disease	9/30
Inhaled steroids (dose, range)	29/30 (358 ± 255, 0–1000 mcg/day)
Long-acting bronchodilators	20/30
Leukotriene antagonists (montelukast)	11/30
Anticholinergic agents (tiotropium)	3/30
Theophylline	1/30
ACT score	22.1 ± 3.3
FEV1 %	75 ± 20.4
FEV1/FVC	0.73 ± 0.1
FEF25-75 %	71 ± 38.2
Peripheral blood eosinophils	0.38 ± 0.31 K/µL
Total serum IgE	198 ± 210 IU/ml

n = 30

**Table 2:** Effect of choline supplementation on ACT scores and spirometric values

	Baseline	3 weeks	6 weeks
ACT (Choline)	22.1 ± 3.3	22.9 ± 2.8	23.4 ± 2
ACT (Placebo)		23.7 ± 1.9	23.2 ± 2.5
FEV1 % (Choline)	75 ± 20.4		75.3 ± 19.3
FEV1 % (Placebo)			76 ± 20
FEV1/FVC (Choline)	0.73 ± 0.1		0.73 ± 0.1
FEV1/FVC (Placebo)			0.73 ± 0.1
FEF25-75 % (Choline)	71 ± 38.2		70 ± 34.2
FEF25-75 % (Placebo)			71.5 ± 34

n = 30

p > 0.86 for all comparisons

Similarly, peripheral blood eosinophil counts and total serum IgE were unaffected by choline supplementation vs. placebo (Table 3). In subjects with lower ACT (≤20, 16.7 ± 3.3, n = 6), lower FEV1 % (<60 %, 46.4 ± 9.2 %, n = 6), or higher eosinophil counts (≥0.6, 0.88 ± 035 K/µL, n = 6) there was also no difference between choline and placebo (Table 4). In subjects with lower serum IgE (≤151 IU/ml, 68.8 ± 46 IU/ml, n = 18), there was a trend for a decrease of the IgE by choline supplementation vs. placebo (56.1 ± 36.9 vs. 69. ± 55.4 IU/ml, respectively), but this did not reach statistical significance (p = 0.078).

**Table 3:** Effect of choline supplementation on peripheral blood eosinophils counts (K/μL) and total serum IgE (IU/ml)

	Baseline	6 weeks
Eosinophils (Choline)	0.38 ± 0.31	0.34 ± 0.27
Eosinophils (Placebo)		0.33 ± 0.27
IgE (Choline)	198 ± 210	206 ± 262
IgE (Placebo)		220 ± 285

n = 30

p > 0.84 for all comparisons

**Table 4:** Effect of choline supplementation in different patient subgroups

	Baseline	6 weeks
ACT ≤ 20 (Choline)	16.7 ± 3.3	21.5 ± 3
ACT ≤ 20 (Placebo)		22.3 ± 2.9
FEV1 % < 60 % (Choline)	46.4 ± 9.2	47 ± 10.3
FEV1 % < 60 % (Placebo)		46.6 ± 6.8
Eosinophils ≥ 0.6 K/µL (Choline)	0.88 ± 0.35	0.62 ± 0.4
Eosinophils ≥ 0.6 K/µL (Placebo)		0.62 ± 0.5

n = 6

p > 0.63 for all comparisons

We found no significant association between eosinophil counts and IgE (Table 5). Similarly, there was no association between eosinophils or IgE and ACT, age, body mass index (BMI), steroid dose, or duration of asthma (Table 5). In subjects with lower ACT scores (16.7 ± 3.3, n = 6), eosinophils and IgE were similar to their counterparts with controlled symptoms (p > 0.22). Eosinophils were similar in subjects treated with higher inhaled steroid dose (≥400 mcg/day, 583 ± 226, n = 13) and subjects on lower doses (p = 0.82). Women had lower baseline ACT scores compared to men (21.1 ± 3.7 vs. 23.6 ± 1.9, respectively, p = 0.02).

**Table 5:** Associations between different parameters at baseline

	R
Eosinophils vs. IgE	0.180
Eosinophils vs. age	0.069
Eosinophils vs. ACT	0.071
Eosinophils vs. BMI	−0.003
Eosinophils vs. inhaled steroid dose	−0.034
Eosinophils vs. duration of asthma	−0.308
IgE vs. age	−0.243
IgE vs. ACT	0.019
IgE vs. BMI	0.154
IgE vs.inhaled steroid dose	−0.145
IgE vs. duration of asthma	0.008

n = 30

p > 0.09 for all associations

None of the subjects reported adverse effects during the course of the study.

### Discussion

In this study, we investigated the effect of choline supplementation in elderly subjects with asthma. This group of patients has been mostly ignored in previous studies of this condition.

Choline supplementation for 6 weeks did not affect asthma symptoms or spirometric values, including in the subgroups of subjects with lower ACT scores and FEV1 values. Similarly, peripheral blood eosinophil counts and total serum IgE were unaffected by choline supplementation. These results suggest that supplementing this methyl donor has no effect on clinical and biologic parameters in elderly asthmatics. We cannot rule out that longer studies using higher doses of choline may have a different outcome. However, an open label study of Indian adults with asthma similarly showed that the same parameters were unaffected by choline chloride supplementation for 6 months [10]. Such study employed higher doses of choline than utilized in our study. In contrast, the same senior author of that study had previously reported that tricholine citrate supplementation for 16 weeks lead to an improvement in asthma symptom scores in small cohorts (10–12 subjects) of adolescent and young adults [11, 12]. One of these two studies was single blinded and the other was open label. It is unclear whether different formulations of choline may affect clinical response.

While blood eosinophil counts are similar in younger and older asthmatics, total serum IgE is significantly lower in elderly than in nonelderly asthmatic subjects [13]. In a study of adults with asthma, antigen specific serum IgE was associated with blood eosinophil counts [14]. We found no association between eosinophils and total serum IgE, or other parameters at baseline.

The results indicating that asthmatic women have lower symptom scores than men are in agreement with a recent study showing an association between female sex and poorer asthma symptom scores [15].

Although choline supplementation is considered fairly safe, and none of our study subjects experienced side effects during the study, one study found that a high dietary intake of choline was associated with an increased risk of colon adenomas in women [16].

Limitations of this study include the relatively small number of subjects, almost exclusively Caucasians and atopic with well controlled asthma. It is possible that including more subjects with uncontrolled symptoms and/or non atopic asthma could yield different results. In addition, it is also possible that choline supplementation may affect biologic markers that were not assessed in our study (e.g., cytokines, periostin).

### Conclusions

In summary, in this pilot study of elderly asthmatics, choline supplementation for 6 weeks did not affect ACT scores, spirometric values, blood eosinophils or serum IgE. These results will require confirmation in subjects with uncontrolled symptoms, in other ethnic groups, and following a longer treatment period with higher dose of choline.

## Abbreviations

BMI: body mass index
ACT: asthma control test
